# Antioxidant and Antimicrobial Activities of Optimized Extract Obtained from Almond Shell Residues

**DOI:** 10.3390/molecules30122614

**Published:** 2025-06-16

**Authors:** Yesuneh Gizaw, María José Benito, Iris Gudiño, María del Carmen Caballero, María de Guía Córdoba, Rocío Casquete

**Affiliations:** 1 Nutrición y Bromatología, Escuela de Ingenierías Agrarias, Universidad de Extremadura, Avd. Adolfo Suárez s/n, 06007 Badajoz, Spain; ygizawch@alumnos.unex.es (Y.G.); igudino@unex.es (I.G.); mcaballerod@unex.es (M.d.C.C.); mdeguia@unex.es (M.d.G.C.); rociocp@unex.es (R.C.); 2 Instituto Universitario de Investigación en Recursos Agrarios (INURA), Universidad de Extremadura, Avd. de la Investigación, 06006 Badajoz, Spain

**Keywords:** almond shell, phenolic extracts, antioxidant, antibacterial, antifungal

## Abstract

The almond shell is a byproduct that contains bioactive compounds such as phenolic compounds and flavonoids, which possess antioxidant and anticancer properties. In this study, these compounds were extracted from the shells of different almond varieties (Lauranne, Marta, Constanti, D01-188, S4017, Vairo) to evaluate their antioxidant, antibacterial, and antifungal activities *in vitro*. The optimal almond varieties for specific bioactivities were identified. Lauranne, due to its elevated phenolic content, exhibited the highest antioxidant capacity. In contrast, S4017, despite its comparatively lower phenolic concentration, demonstrated the most potent antibacterial activity. Constanti, characterized by its high content of both phenolic and flavonoid compounds, is well-suited for promoting overall health and enhancing food preservation, while Vairo exhibits notable antifungal activity against specific fungal species. These findings suggest that selecting almond varieties based on their bioactive compound profiles can enhance their health benefits and applications in the food and pharmaceutical industries.

## 1. Introduction

The almond tree is one of the most important perennial crops in the Mediterranean basin. Some of the most prominent varieties in Spain offer numerous benefits, although they also present certain limitations. Varieties such as Marta, Lauranne, Constanti, and Vairo are particularly notable for their high cumulative yield of shelled almonds and almonds with a high oleic/linoleic acid ratio [1].

The edible part consists solely of the almond, which constitutes only a small percentage of the whole nut. On average, the edible part of almonds is 24% of the total nut; thus, 76% is waste. Almond shells can represent the most abundant component among the byproducts of almond processing, considering that the shell percentage in one kilogram of almonds can reach up to 80% for Mediterranean cultivars [2]. The shell is mainly composed of cellulose, hemicellulose, and lignins, which are responsible for the hardness and resistance of the shell, making it a good fibrous material that can be exploited for cellulose extraction and subsequent use in the design of biodegradable and eco-friendly materials [3,4]. They have also been used as fuel through combustion or as animal feed [5]. However, interest in these by-products has increased due to their bioactive compounds, primarily polyphenols, which have beneficial properties that make them very interesting for use as new ingredients in the food, cosmetic, and pharmaceutical industries. Several scientific studies support the high content of phenolic compounds in almond shells; however, the quantities and number of total phenolic compounds detected vary greatly depending on the study, the extraction protocol applied, the quantification units used, the standards employed to express final concentrations, and the detection method chosen to analyze phenolic content [5].

Some conventional methods used for the extraction of phenolic compounds from byproducts of different plant matrices include distillation, solvent extraction, cold compression, electric energy extraction, and ultrasound-assisted extraction, among others [6]. Common solvents used for polyphenol extraction include ethanol, water, n-hexane, ethyl acetate, and acetone, with the combination of ethanol and water assisted by ultrasound being one of the best combinations, as it leaves no residue for subsequent use in food [7,8].

The almond shell by-product has been studied as a bioconservant and even as a pharmaceutical product [9]. Bisignano et al. [10] investigated the polyphenols in almond skin for their antibiotic properties, obtaining promising results. However, there are few studies on the antifungal activity of phenolic extracts compared to bacterial activity. To date, only a few extracts or compounds of plant origin have demonstrated antifungal activity against human and animal pathogens. The most studied are those obtained from different matrices of *Vitis vinifera* L. [11].

Therefore, the aim of this work was to extract and characterize the phenolic compounds from the shells of different almond varieties and study their antioxidant and antimicrobial activities for the development of new value-added products that contribute to reducing environmental impact and improving the sustainability and competitiveness of the almond industry.

## 2. Results

### 2.1. Phenolic Compounds and Flavonoids of Extracts Obtained from the Shells of Different Almond Varieties

Table 1 presents the total phenolic compounds and flavonoids (mg·L^−1^) extracted from different almond varieties. It is noteworthy that the values of phenolic compounds and flavonoids vary significantly among the different varieties analyzed, suggesting that the chemical composition of the extracts may be influenced by genetic and environmental factors.

Regarding the phenolic compound content, the varieties Constanti and Lauranne exhibited the highest values (*p* < 0.05) compared to the other varieties. Conversely, S4017 and Vairo showed the lowest content (*p* < 0.05). The significant differences in phenolic compound content among the varieties could have implications for their antioxidant potential and health benefits.

In terms of flavonoids, Constanti, D01-188, and S4017 and Vairo have the highest levels, between 3520.04 mg·L^−1^ and 3036.43 mg·L^−1^ (Table 1). In contrast, Marta presents the lowest flavonoid content with 1344.62 mg·L^−1^. Similar to phenolic compounds, the differences in flavonoid levels are also statistically significant, suggesting that some varieties may be richer in these bioactive compounds than others.

The results highlight the importance of selecting specific almond varieties to maximize the content of phenolic compounds and flavonoids, which could be beneficial for applications in the food industry and health promotion.

Table 2 presents the results of the identification and quantification of bioactive compounds in almond extracts, analyzed using the HPLC-UV-ESI-MS/MS technique. The identified compounds are categorized into three main groups: phenols, acids, and others, with their corresponding retention times (Rt) and fragments obtained in the MSn mass spectrometry experiments.

Overall, a notable variability in the concentration of these compounds is observed among the different almond varieties studied. In addition, statistical analysis indicates that there are significant differences (*p* < 0.05) in the concentrations of the compounds in the different extracts, suggesting that each almond variety possesses a unique profile of bioactive compounds.

Regarding phenolic compounds, several phenolic acids, flavonoids, and their derivatives stand out. Hydroxycinnamic acid (peak 1) is present in higher concentrations in the Marta variety (164.22), trans-p-coumaric acid (peak 2) has its highest concentration in Constanti (102.23), while S4017 and Vairo have considerably lower levels. A particularly relevant compound is 4,5-dicaffeoylquinic acid (peak 5), which shows high concentrations in S4017 (320.35) and Vairo (290.10), and 3,6,2′,3′-Tetrahydroxyflavone in Lauranne and Marta (172.82 and 183.46 respectively). Marta was also highlighted for its concentration in Procyanidin dimer B [Epi]catechin dimer (367.8).

In the category of organic acids, 2-Methylsuccinic acid (peak 11) is found in notably high concentrations in S4017 (232.60), while gluconic acid (peak 12) and quinic acid (peak 13), which is more prominent in Constanti (156.31 and 261.79) followed by D01-188 (80.48 and 190.82).

The analysis of other compounds shows that some bioactive compounds, such as ursolic acid (peak 20) and corosolic acid (peak 19), are present in higher concentrations in Vairo and S4017 (150.93 and 142.74, respectively). Both compounds have recognized anti-inflammatory and anticancer properties, which could give the Vairo and S4017 varieties an advantage in terms of health benefits and therapeutic applications.

In summary, the results underscore the variability in the composition and concentration of compounds among the different almond variety extracts. These findings suggest that the bioactive compound profile of each almond variety could have important implications for their use in the food and pharmaceutical industries.

### 2.2. Antioxidant Activity of Extracts Obtained from the Shells of Different Almond Varieties

The results of antioxidant activity measured by the 2,2-diphenyl-1-picrylhydrazyl (DPPH) radical scavenging assay and the 2,2′-azinobis-(3-ethylbenzothiazoline)-6-sulfonic acid (ABTS) radical scavenging assay, expressed in mg of Trolox per 100 g of extract, are shown in Table 3. It is noteworthy that the Lauranne variety stands out with the highest values in both measurements, with 197.97 mg in DPPH and 304.14 mg in ABTS. This suggests that this variety has superior antioxidant potential compared to the others. In contrast, the S4017 variety shows the lowest values, with only 16.31 mg in DPPH and 127.85 mg in ABTS, indicating significantly lower antioxidant capacity.

The results demonstrate that antioxidant activity was directly related to the concentration of total phenolic compounds in the samples.

### 2.3. Antimicrobial Activity of Extracts Obtained from the Shells of Different Almond Varieties

Regarding the results of antimicrobial activity, analysis was conducted against bacteria and molds (Table 4).

Table 4 presents the results of a two-way ANOVA multivariate analysis conducted to evaluate the antibacterial activity (% inhibition) of almond shell extracts from different varieties against six bacterial strains (*Bacillus cereus*, *Staphylococcus aureus*, *Escherichia coli*, *Salmonella choleraesuis*, *Listeria innocua* and *Listeria monocytogenes*). The two factors considered in the analysis were extract type (E; Constanti, D01-188, Lauranne, Marta, S4017, and Vairo) and concentration (C; 1600, 800, and 400 mg L^−1^), and their individual effects, as well as their interaction (E × C), were tested for statistical significance across the different bacteria.

Regarding the extracts, it was observed that all of them exhibited activity against the tested bacteria. The S4017 variety extract showed the highest inhibition capacity compared to the other varieties for all the bacteria studied (*p* < 0.05; Table 4), with inhibition percentages exceeding 94%.

These results indicate that the activities of the extracts depend on their composition, as the S4017 extract, which had the lowest phenolic compound content (Table 1), showed the highest inhibition capacity. As indicated by the results in Table 4, antibacterial activity is influenced by the concentration of compounds present in the extracts for all bacteria studied. Inhibition greater than 90% was achieved in *B. cereus*, *S. aureus*, and *S. choleraesuis* using the 1600 mg·L^−1^ concentration of the extracts. A progressive decline in antibacterial efficacy was observed with decreasing extract concentrations, with inhibition levels dropping to approximately 28% and 37% against *B. cereus* and *L. monocytogenes*, respectively.

The results also provide valuable information on the antibacterial activity of different almond extracts. The S4017 variety appears to be the most promising, while the concentration of the extracts plays a crucial role in their effectiveness. These findings could have important implications for the development of new antimicrobial agents from natural sources such as almonds by-products.

**Figure 1:**
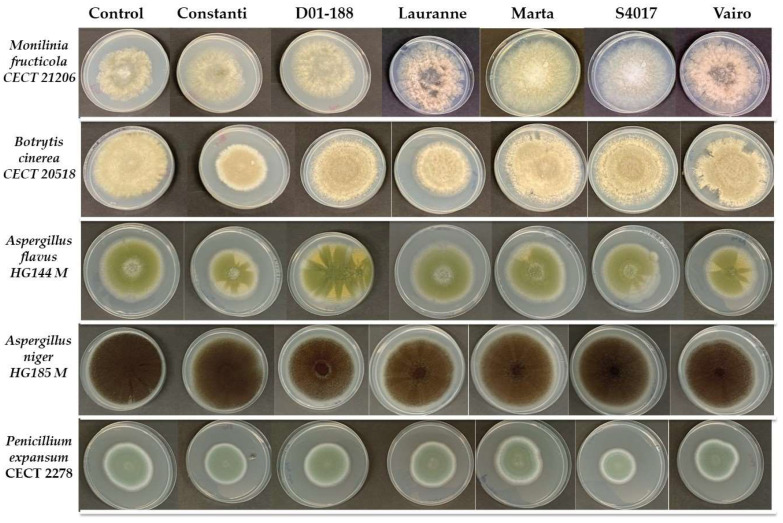
Potato dextrose agar plates of the tested mold strains’ growth obtained after 7 days of incubation at 25 °C in the presence of the phenolic extracts (5000 mg·L^−1^) obtained from different almond shells extracts.

On the other hand, Figure 1 and Table 5 present the growth (in mm) of different mold strains after 7 days of incubation at 25 °C in the presence of extracts at a concentration of 5000 mg·L^−1^. Five fungal species were evaluated: *Monilinia fructicola*, *Botrytis cinerea*, *Aspergillus flavus*, *Aspergillus niger* and *Penicillium expansum.*

The results show that in the control, without the presence of extracts, the strains achieved considerable growth, with values ranging from 56.70 mm in *M. fructicola* to 80.90 mm in *A. niger*. This indicates that the experimental conditions were optimal for mold development. However, in the presence of the extracts, significant reductions in the growth of some strains were observed compared to the control, suggesting that certain compounds present in the extracts exerted an inhibitory effect.

The effects of the extracts varied according to the fungal species evaluated. In the case of *B. cinerea*, growth was significantly lower with the extracts Constanti (60.75 mm) and Lauranne (60.67 mm), while the extract S4017 showed the opposite effect, promoting the growth of the strain to 83.25 mm. On the other hand, *A. flavus* showed greater sensitivity to the extracts Constanti and Vairo, with growth values of 52.50 mm and 54.67 mm, respectively, compared to the control (66.30 mm). In contrast, *A. niger* showed a significant reduction with most extracts, although values were close to the control.

*P. expansum* was the species most affected by the extracts, showing significant reductions in growth with Constanti, D01-188, S4017, and Vairo, with values ranging from 44.50 mm to 49.83 mm. This decrease suggests that the extracts may contain compounds with a fungistatic or fungicidal effect against this particular species.

In conclusion, the evaluated extracts show a variable effect on the growth of fungal strains, with some, such as Constanti and Vairo, demonstrating greater inhibitory capacity. However, the lack of effect in some species and even the increase in growth under certain conditions suggest that the antifungal activity of these extracts depends both on their chemical composition and the specific susceptibility of each mold.

### 2.4. Multivariate Analysis of the Parameters Studied

**Figure 2:**
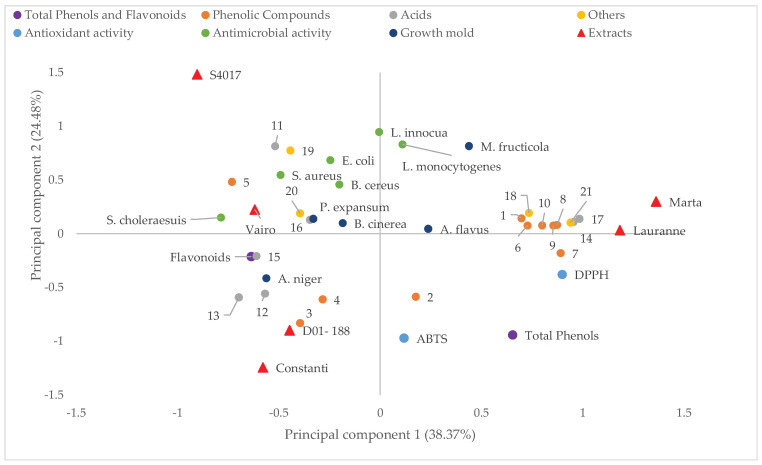
Projection on a plane of principal components 1 and 2 of the principal component analysis of the studied parameters of extracts obtained from the shells of different almond varieties. Compounds identified (1–21, Table 2). Antioxidant activity by two methods (DPPH and ABTS). Antibacterial activity (% inhibition) (*B. cereus*, *S. aureus*, *E. coli*, *S. choleraesuis*, *L. innocua*, and *L. monocytogenes*). Growth (mm) of molds in the presence of the extracts (*M. Fructicola*, *B. cinérea*, *A. flavus*, *A. niger* and *P. expansum*).

Figure 2 presents the principal component analysis (PCA), projecting the samples and variables onto the plane defined by principal component 1 (PC1) and principal component 2 (PC2), which explain 38.37% and 24.48% of the total variance, respectively. PC1 is primarily associated with antioxidant activity measured by the DPPH method, located on the right side of the plot, where almond cultivars such as Marta and Lauranne are positioned, indicating a higher antioxidant capacity in their extracts.

Moreover, the position of phenolic compounds such as Dihydroquercetin (peak 6), 3,6,2′,3′-Tetrahydroxyflavone (peak 7), and Procyanidin B/Epicatechin dimer (peak 10) suggests their potential contribution to the antioxidant activity observed in these cultivars.

On the other hand, PC2 differentiates the samples based on the antimicrobial activity of the extracts (*B. cereus*, *S. aureus*, *E. coli*, *S. choleraesuis*, *L. innocua*, and *L. monocytogenes*), with the cultivar S4017 showing a positive correlation with this activity. The S4017 cultivar exhibits a higher concentration of compounds such as 2-Methylsuccinic acid (peak 11) and Corosolic acid (peak 19), which may be involved in the inhibition of bacterial growth.

At the lower part of the plot, a grouping of flavonoid content is observed along with compounds such as p-Hydroxybenzoic acid (peak 3) and p-Coumaric acid (peak 4), which are associated with the extracts from the Constanti and D01-188 cultivars.

The different almond cultivars (represented by red triangles) are dispersed throughout the plane, reflecting their distinct characteristics in terms of antioxidant activity, antibacterial potential, and possible influence on mold growth.

In summary, this principal component plot provides a visual representation of the complex relationships between the almond cultivars studied and the various parameters analyzed, enabling the identification of trends and potential correlations between the composition of the extracts and their biological activities.

## 3. Discussion

Our results highlight the relevance of screening specific almond varieties to optimize the phenolic compound and flavonoid content, both of which may be beneficial for food industry and health promotion applications. It has been proven that almonds are rich sources of phenolic compounds, which possess healthy beneficial properties.

Moreno Gracia et al. [17] studied the total amount of polyphenols, flavonoids, and proanthocyanidins, as well as the antioxidant capacity of different almond varieties from Spain. The results highlight the differences in antioxidant content, which add value to the quality of the fruit. Also, it has been shown that the genotype can strongly influence the antioxidant capacity and total phenolic compounds. Additionally, almond shells or husks have also revealed significant amounts of total phenols and flavonoids, which possessed moderate antioxidant activity [18,19].

The identified compounds have been referenced as contributing to human health through their antioxidant, anti-inflammatory, and anticancer properties. The variability in the concentration of these compounds among different almond varieties suggests that the selection of specific varieties could be crucial to maximize the desired functional benefits, which is in line with what has been stated above. The Marta variety stands out for its high content of hydroxycinnamic acid and 4,5-dicaffeoylquinic acid, phenolic compounds whose antioxidant properties have been widely documented in studies with nuts by-products [20]. This latter compound, particularly abundant in Marta, has demonstrated the ability to modulate inflammatory pathways such as NF-κB and MAPK in cellular models [21], suggesting its potential as a functional ingredient. On the other hand, Constanti presents the highest level of trans-p-coumaric acid, a phenol whose content varies significantly according to environmental and genetic factors, as observed in analyses of Mediterranean varieties’ shells [22].

In the flavonoid group, the presence of 3,6,2′,3′-tetrahydroxyflavone in Lauranne and Marta stands out, a compound rarely reported in almonds but with antioxidant activity comparable to that of isoflavones *in vitro* studies [23]. The dimeric procyanidin B in Lauranne coincides with previous findings identifying these condensed tannins as major components in almond skins, where they contribute to the oxidative stability of the fruit [24].

The profile of organic acids reveals distinctive patterns: S4017 accumulates 232.60 of 2-methylsuccinic acid, a metabolite linked to oxidative stress processes in plants, while Constanti shows a predominance of quinic acid, a key compound in the synthesis of aromatic esters during roasting. These results are consistent with studies associating quinic acid with preservative properties in food matrices [25].

The triterpenoid compounds of Vairo and S4017 are also of great relevance, where ursolic and corosolic acids exhibit concentrations comparable to those reported in standardized extracts of medicinal herbs [26]. Both compounds have demonstrated antiproliferative activity against breast and colon cancer cell lines through the induction of apoptosis [27].

These compositional variations among varieties underscore the importance of genetic selection for specific applications. While Constanti could be prioritized for food preservation processes due to its acidifying profile, S4017 and Vairo emerges as an ideal candidate for multifunctional extracts.

Our results showed that the antioxidant activity was directly related to the concentration of total phenolic compounds in the samples, with the Lauranne variety being the sample with the highest antioxidant activity and the highest concentration of compounds.

These findings are consistent with those reported by other researchers [17,18,19,28]. Over the past decade, there has been growing interest in antioxidants due to their health-promoting properties, which are associated with the prevention of degenerative diseases [29]. An antioxidant prevents the oxidation process, the initial step in the development of degenerative diseases, cancer, and many others. Additionally, antioxidants are also related to antimicrobial activity, thereby contributing to food safety and extending shelf life [30]. The crucial key in disease prevention is the action these antioxidants exert after consumption.

Based on these results, we can conclude that the extracts from the Lauranne, Constanti, and Marta almond varieties stand out for their high content of phenolic compounds with antioxidant activity.

On the other hand, the findings offer valuable insights into the antibacterial properties of various almond extracts. The S4017 variety emerges as the most promising, with the concentration of the extracts being a critical factor in their effectiveness. Various authors have confirmed this, and when analyzing the beneficial effects of phenolic compounds, they assert that several factors must be considered due to their clear influence. The concentration and content of phenolic compounds directly affect the activity [31,32].

Musarra-Pizzo et al. [33] tested the antibacterial effect of a mixture of polyphenols present in natural almond skin. The antimicrobial potential was evaluated against clinical strains of *Staphylococcus aureus*, demonstrating that the epicatechin and catechin compounds present in the extract showed greater activity against *S. aureus* ATCC 6538P but were not active against all other strains. Similarly, Mandalari et al. [34] evaluated the antimicrobial properties of flavonoid-rich fractions derived from natural and blanched almond skins, the latter being a byproduct of the almond processing industry. Almond skin extracts were tested against Gram-negative bacteria (*Escherichia coli*, *Pseudomonas aeruginosa*, *Salmonella enterica*, and *Serratia marcescens*) and Gram-positive bacteria (*Listeria monocytogenes*, *Enterococcus hirae*, *Staphylococcus aureus,* and *Enterococcus durans*). It was observed that almond skin fractions exhibited antimicrobial activity against the Gram-positive bacteria *L. monocytogenes* and *S. aureus* in the range of 250–500 μg/mL and also showed antimicrobial potential against the Gram-negative bacterium *Salmonella enterica*.

Bouaziz et al. [35] studied polyphenol extracts and volatile compounds from almonds and investigated their antibacterial activities against 10 pathogenic strains, showing maximum growth inhibition when using 50 mg of solvent extract against *Enterobacter* spp., *Salmonella typhimurium*, *Listeria monocytogenes*, *Micrococcus luteus,* and *Bacillus subtilis.*

In our study, ursolic and corosolic acids were found in high concentrations in the Vairo and S4017 varieties. These compounds have shown efficacy against key bacterial pathogens, *Staphylococcus aureus* and *Escherichia coli*, by altering bacterial cell membrane permeability [36]. Hydroxycinnamic acid derivatives, particularly abundant in Marta, also exhibit antimicrobial properties. The 4,5-dicaffeoylquinic acid (4,5-diCQA) identified in cultivars S4017 and Vairo may compromise microbial membrane integrity, thereby limiting pathogen invasion. Previous studies have reported that phenolic extracts containing chlorogenic acid, ferulic acid, and p-coumaric acid can disrupt microbial membrane structures, leading to cytoplasmic leakage and cellular dysfunction, primarily through membrane integrity loss and protein denaturation [37,38]. These compounds are biochemically related to 4,5-diCQA, as it is a diester derivative of chlorogenic acid with two caffeoyl groups. Moreover, both p-coumaric and ferulic acids serve as biosynthetic precursors of caffeic acid, thus establishing an indirect link to 4,5-diCQA. Furthermore, trans-p-coumaric acid, which is abundant in the Constanti cultivar, may play a role in inducing membrane hyperpolarization and compromising cellular integrity in *Staphylococcus aureus*. This potential mechanism is supported by previous findings demonstrating similar effects caused by chlorogenic acid [38], a compound synthesized from caffeic acid. Given that caffeic acid is biosynthetically derived from trans-p-coumaric acid, this metabolic connection suggests that the antimicrobial activity of trans-p-coumaric acid could, at least in part, stem from its role as a precursor in the chlorogenic acid biosynthetic pathway. These activities are also enhanced in matrices rich in organic acids like quinic acid, abundant in Constanti, which contributes to the stability of antimicrobial phenols in food systems [25]. Flavonoids and procyanidins in Lauranne and Marta have shown inhibitory effects against foodborne pathogens. Procyanidins isolated from *Rhododendron formosanum* leaves showed strong antibacterial activity against *Staphylococcus aureus*, suggesting their potential for pharmacological applications [39].

Although various studies have demonstrated the antibacterial activity of phenolic extracts obtained from almonds with good results, it is also noteworthy that the activities have been quite varied. This is because each study used different almond varieties obtained from different locations, resulting in varying concentrations and compositions of compounds from one study to another.

These results could significantly impact the development of new antibacterial agents derived from natural sources, such as almond by-products.

Regarding antifungal activity, the evaluated extracts exhibit varying effects on the growth of fungal strains. Some, like Constanti, show greater inhibitory capacity. However, the lack of effect on certain species and even the increase in growth under specific conditions suggest that the antifungal activity of these extracts depends both on their chemical composition and the specific susceptibility of each mold. There is less information in the literature regarding activity against fungi, and more specifically against spoilage and pathogenic molds. Various authors have tested different phenolic extracts for antifungal activity. Mendoza et al. [40] evaluated the phenolic compounds of grape pomace extracts for their *in vitro* antifungal activity against the phytopathogenic fungus *Botrytis cinerea*. They observed varying activity against *B. cinerea* depending on the extraction method used. Hajji-Hedfi et al. [41] analyzed the phytochemical components of the aqueous extract of *Capsicum annuum* seeds and evaluated its *in vitro* antifungal activity at different concentrations (10, 20, 30, and 60%). They found that the aqueous extract of *C. annuum* seeds combined with salicylic acid had positive effects in terms of inhibiting *B. cinerea*, concluding that it is a promising and environmentally friendly alternative to chemical fungicides for sustainable agriculture under climate change. Rodrigues et al. [42] evaluated the effects of phenolic extracts from tomato samples against the strain *Penicillium expansum* CCT 7549. Phenolic extracts from conventionally managed tomatoes were effective in inhibiting *P. expansum*, which they attributed to the presence of caffeic acid in these extracts. However, *in vivo* studies on the fruit did not inhibit growth or mycotoxin production. Jahani et al. [43] tested the inhibitory effects of essential oils of eucalyptus, galbanum, thyme, and clove against *Aspergillus niger* at various concentrations (0, 200, 400, 600, and 800 μL L^−1^) *in vitro* and *in vivo*. In vitro results showed that *A. niger* growth was completely inhibited by the application of clove and thyme oils at high concentrations. *In vivo* results indicated that only fruits treated with thyme oil exhibited the best antifungal effects. The essential oils had a high concentration of various phenolic compounds responsible for this activity. Finally, Liu et al. [44] investigated the antifungal activity against *Aspergillus flavus* of citrus peel extracts prepared with food-grade solvents (hot water or ethanol). Ethanol extracts of mandarin peel (*Citrus reticulata*) inhibited the mycelial growth of *A. flavus* (39.60%) more effectively than those of orange (32.31%) and lemon (13.51%) after 7 days of incubation. They concluded that citrus peels are promising biological resources of antifungal agents with potential applications in the food industry and other industries.

The reviewed studies demonstrate that phenolic extracts and other natural compounds have significant potential as antifungal agents. The efficacy of these extracts varies depending on the extraction method, the concentration used, and the combination with other compounds agreeing with the results of this study. These findings underscore the importance of continuing to research and develop sustainable and environmentally friendly alternatives to chemical fungicides, especially in the context of climate change and the need for more sustainable agricultural practices.

## 4. Materials and Methods

### 4.1. Plant Material

The shells of different varieties of almonds (Marta, Lauranne, Constanti, D01-188, S4017, Vairo) from an industrial plots located in the municipality of Seville, Spain, were used for this work.

### 4.2. Bacterial and Fungal Strains

For this study, eight strains from the Spanish Type Culture Collection were used: *Bacillus cereus* CECT 131, *Staphylococcus aureus* CECT 976, *Escherichia coli* CECT4267, *Salmonella choleraesuis* CECT 4395, *Listeria innocua* CECT 910, *Listeria monocytogenes* CECT 911, *Botrytis cinerea* CECT 20518, and *Penicillium expansum* CECT 2278. Additionally, three strains from the collection of the CAMIALI group from the University of Extremadura, Spain, were used: *Aspergillus flavus* HG 144 M, *Monilinia fructicola* 362, and *Aspergillus niger* HG 185 M.

### 4.3. Extraction of Total Phenolic Compounds and Flavonoids

Bioactive compounds of interest were extracted from the by-products in triplicate using ultrasound-assisted extraction with 80% ethanol (*v*/*v*) as the solution [7]. For this, 10 g of dry shells was mixed with 60 mL of ethanol (80%) and subsequently placed in an ultrasonic bath (40 kHz) at 45 °C for one hour. The extract was then filtered through filter paper, and another 60 mL of the extraction solution was added to the remaining solids, repeating the previously described process. After the second filtration, the combined extracts were evaporated to remove ethanol, yielding an aqueous extract. Finally, the obtained extract was stored at −20 °C until further analysis.

### 4.4. Determination of Total Phenolic Compounds and Flavonoids Content

Determination of total phenolic compounds content was carried out using the Folin-Ciocalteu colorimetric method. This method uses a mixture of sodium tungstate and molybdate in phosphoric acid that reacts with phenolic compounds capable of reducing the reagent, producing a blue coloration that can be measured with a spectrophotometer at 760 nm. Gallic acid was used as the standard. For the method, 25 mL flasks were used for each sample. To each flask, 10 mL of MilliQ water, 0.5 mL of the corresponding sample, 1 mL of Folin–Ciocalteu reagent, and 2 mL of a saturated sodium carbonate (Na_2_CO_3_) solution were added. The volume was adjusted to 25 mL by adding MilliQ water and the contents were mixed vigorously. The flasks were incubated at room temperature in the dark for 60 min, after which the absorbance was measured at 760 nm. The total phenolic content of each extract was determined using a calibration curve prepared with gallic acid. The results are expressed in mg L^−1^.

The measurement of flavonoids was carried out following the method described by [45]. For this purpose, 3 mL of methanol, 1 mL of the sample, 0.2 mL of aluminum chloride (10% *w*/*v*), 0.2 mL of potassium acetate (1M), and finally 5.6 mL of distilled water were added to test tubes. The tubes were then incubated in the dark for 30 min. Finally, the absorbance was measured with a spectrophotometer at 415 nm. The flavonoid content in the sample extracts was quantified using a quercetin calibration curve. The results are expressed in mg L^−1^.

### 4.5. Separation and Identification of Bioactive Compounds

The compounds present in each extract were identified by diluting them in methanol to a final concentration of 100 ppm. All solvents were LC-MS grade. The samples were filtered through a syringe filter (0.45 μm) and injected into an HPLC system coupled to a mass spectrometer (HPLC-QTOF Agilent Model G6530, Agilent Technologies, Palo Alto, CA, USA) equipped with a C18 column (4.6 × 150 mm, 4.8 μm). The detection and identification of bioactive compounds were performed using a quadrupole time-of-flight tandem mass analyzer (Q-TOF) with an electrospray ionization (ESI) source in negative mode. The instrument operated in negative-ion mode, performing a full scan across the mass range of *m*/*z* 100 to 1700. The gas flow was 11 mL/min at 280 °C (nebulizer 35 psi). Gradient elution was carried out with a mixture of hydrocyanic acid/water (5:95, *v*/*v*) as solvent A and hydrocyanic acid/water/formic acid (95:4.9:0.1, *v*/*v*/*v*) as solvent B, with a flow rate of 0.350 mL/min. The solvent gradient started with solvent B/solvent A (5:95, *v*/*v*), gradually reaching 90:10 (*v*/*v*) at 15 and 20 min, before returning to the initial conditions for the final 10 min. Tentative identification of bioactive compounds was carried out by comparison with the MassBank database (https://massbank.eu/MassBank/, accessed on 29 April 2025), and their relative abundances were expressed as peak areas in arbitrary units (a.u.).

### 4.6. Antioxidant Determination of the Phenolic Extracts

To determine antioxidant capacity, two methods were employed: the 2,2-diphenyl-1-picrylhydrazyl (DPPH) method and the ability to scavenge the 2,2′-azinobis (3-ethylbenzothiazoline-6-sulfonic acid) (ABTS) radical. Trolox was used as the standard, and the results are expressed in mg of Trolox/100 g of extract.

The DPPH method was performed according to the procedure of [46]. In a cuvette, 2950 μL of DPPH reagent and 50 μL of sample were added and allowed to react for 30 min at room temperature in darkness. After this period, measurements were taken with a spectrophotometer at a wavelength of 515 nm.

The ABTS method was performed according to the method of [47] with slight modifications. Briefly, 1 mL of ABTS radical cation was placed in a spectrometer cuvette along with 20 μL of sample. The initial absorbance value at 730 nm was measured and compared with the absorbance obtained after 20 min of reaction.

### 4.7. Antimicrobial Activity Determination

To determine the antibacterial activity of the extracts obtained from different samples, the growth capacity in liquid medium was analyzed. The culture medium used for the bacteria was brain heart infusion (BHI) broth, prepared according to the manufacturer’s instructions. The bacteria were grown at 37 °C for 24 h. Subsequently, a 2% (*v*/*v*) suspension of each microorganism at 10^5^ CFU/mL was inoculated into each well of a microtiter plate, along with different concentrations of the various extracts. Once the microtiter plate was filled with different concentrations, negative control, and positive control, it was placed in the plate reader for 24 h at 37 °C. During this time, absorbance readings at 560 nm were automatically collected every hour, allowing observation of the growth or inhibition of the microorganisms. The results were expressed as the percentage inhibition of the extract against each microorganism.

To determine the antifungal activity of the different extracts, the growth capacity in solid medium was analyzed. The medium used was Potato Dextrose Agar (PDA), which is specific for analyzing molds, and was prepared according to the manufacturer’s instructions. For the inoculation of each mold, solutions of 1.5 mL of 106,106 spores/mL were prepared from molds grown on PDA plates using a Neubauer chamber. In each PDA plate, 100 microliters of sample (extract 5000 mg L^−1^) were spread with a glass rod, and 5 µL of the spore suspension of each mold were added. Lower extract concentrations were tested but did not exhibit any inhibitory effect. The inoculation of the spore suspension of each mold was used as a positive control, and water was used as negative control. The molds were incubated in an oven at 25 °C for 7 days, and their diameter was measured horizontally and vertically over this period. The results were expressed in millimeters of growth (mm).

### 4.8. Statistical Analysis

For the statistical analysis of the mean values obtained from the conducted analyses, the SPSS for Windows 22.0 program (SPSS Inc., Chicago, IL, USA) was used. Significant differences and homogeneous groups of means were established through one-way analysis of variance (ANOVA), while a two-way ANOVA was applied for the evaluation of antimicrobial activity. When the interaction effect was significant (*p* < 0.05), a mean comparison test was performed using the TUKEY method. Principal component analysis (PCA) was performed on the correlation matrix of the variables.

## 5. Conclusions

The study identifies the optimal almond varieties for specific bioactivities. Lauranne, with its high phenolic content, demonstrates superior antioxidant activity; S4017, despite lower phenolic levels, exhibits the most effective antibacterial activity; Constanti, with high levels of both phenolic and flavonoid compounds, is ideal for overall health benefits and food preservation; and Vairo, which shows significant antifungal properties against certain fungal species. These findings suggest that selecting almond varieties based on their bioactive compound profiles can enhance their health benefits and applications in the food and pharmaceutical industries.

## Figures and Tables

**Table 1:** Total phenolic compounds and flavonoids (mg·L^−1^) in the extracts obtained from the shells of different almond varieties.

	Total Phenols			Flavonoids		
Extract	Mean		TD *	Mean		TD
Constanti	6883.35	±	496.76 ^ab^	3405.80	±	569.93 ^a^
D01-188	5375.22	±	454.32 ^b^	3520.04	±	55.82 ^a^
Lauranne	7318.20	±	150.11 ^a^	2099.35	±	9.20 ^b^
Marta	6168.95	±	322.20 ^b^	1344.62	±	194.37 ^c^
S4017	3332.28	±	166.49 ^c^	3350.67	±	29.93 ^a^
Vairo	3852.87	±	522.05 ^c^	3036.43	±	9.36 ^a^

* TD: typical deviation; ^abc^ mean values with different numbers indicate statistical differences (*p* < 0.05) between extracts.

**Table 2:** Identification and quantification in arbitrary area units of the main compounds from almond shell extracts analyzed by HPLC-UV-ESI-MS/MS.

Peak	Rt (min)	[M − H]^−^	HPLC-ESI(-)-MS^n^ Experiment *m*/*z*	Compounds Identified	Constanti	D01-188	Lauranne	Marta	S4017	Vairo
Phenolic Compounds										
1	3.18	581	393; 525; 582; 637	Hydroxycinnamic acid ^1^	25.6 ^b^	19.3 ^b^	28.64 ^b^	164.22 ^a^	22.69 ^b^	19.97 ^b^
2	13.03	163	135; 145; 377	Trans-p-coumaric acid ^2^	102.23 ^a^	25.3 ^b^	92.49 ^a^	8.26 ^b^	7.31 ^b^	6.99 ^b^
3	16.39	137	377	p-Hydroxy-benzoic acid ^3^	87.31 ^a^	57.20 ^a^	0.00 ^b^	0.00 ^b^	0.00 ^b^	0.00 ^b^
4	17.08	163	164; 271	p-Coumaric acid ^2^	162.13 ^a^	0.00 ^b^	0.00 ^b^	0.00 ^b^	0.00 ^b^	0.00 ^b^
5	21.32	515	-	4,5-Dicaffeoylquinic acid ^1^	0.00 ^b^	220.02 ^a^	0.00 ^b^	0.00 ^b^	320.35 ^a^	290.10 ^a^
6	24.04	303	116; 250; 176	Dihydeoquercetin ^2^	56.12 ^b^	48.14 ^b^	60.48 ^b^	202.47 ^a^	39.88 ^b^	49.75 ^b^
7	26.05	149	121	3,6,2′,3′-Tetrahydroxyflavone ^1^	132.98 ^a^	70.16 ^b^	172.82 ^a^	183.46 ^a^	65.27 ^b^	63.09 ^b^
8	26.17	479	480	Myricetin glucoside ^4^	0.00 ^c^	0.00 ^c^	34.07 ^a^	15.55 ^b^	0.00 ^c^	0.00 ^c^
9	13.96	475	182; 209; 210; 476	Dimethyl ellagic acid pentoside ^5^	0.00 ^c^	0.00 ^c^	27.36 ^a^	11.38 ^b^	0.00 ^c^	0.00 ^c^
10	20.20	577	149; 193	Procyanidin dimer B [Epi]catechin dimer ^6^	45.32 ^b^	54.14 ^b^	99.22 ^a^	367.8 ^b^	23.69 ^b^	24.82 ^b^
Acids										
11	2.76	131	104	2-Methylsuccinic acid ^1^	0.00 ^c^	0.00 ^c^	0.00 ^c^	0.00 ^c^	232.60 ^a^	49.93 ^b^
12	2.92	195	103; 129; 357	Gluconic acid ^1^	156.31 ^a^	80.48 ^b^	0.00 ^c^	0.00 ^c^	57.89 ^b^	0.00 ^c^
13	3.02	191	109; 192	Quinic acid ^1^	261.79 ^a^	190.82 ^b^	0.00 ^e^	0.00 ^e^	107.79 ^c^	57.45 ^d^
14	3.07	100	112; 128; 154; 214	4-(Pentanamidomethyl) benzoic acid ^1^	0.00 ^c^	0.00 ^c^	198.68 ^a^	142.07 ^b^	0.00 ^c^	0.00 ^c^
15	3.20	133	115; 175	Malic acid ^1^	38.30 ^b^	228.69 ^a^	0.00 ^c^	0.00 ^c^	65.75 ^b^	197.41 ^a^
16	18.05	487	488; 489	Pygenic acid C ^1^	11.27 ^b^	0.00 ^c^	0.00 ^c^	0.00 ^c^	14.68 ^b^	261.39 ^a^
17	18.64	119	141; 437; 453	2,4,6-Trimethylbenzoic acid ^1^	0.00 ^c^	0.00 ^c^	201.76 ^b^	253.73 ^a^	0.00 ^c^	0.00 ^c^
Others										
18	15.85	300	132; 301; 344	N-Acetyl-alpha-D-glucosamine 1-phosphate ^1^	3.65	4.78	5.44	15.29	4.9	3.15
19	21.00	471	472; 473	Corosolic acid ^1^	0.00 ^b^	0.00 ^b^	0.00 ^b^	0.00 ^b^	142.74 ^a^	0.00 ^b^
20	25.89	455	456	Ursolic acid ^1^	8.95 ^c^	0.00 ^c^	0.00 ^c^	0.00 ^c^	21.43 ^b^	150.93 ^a^
21	25.95	184	157	N4-Acetylsulfadiazine^1^	0.00 ^c^	0.00 ^c^	20.57 ^a^	13.36 ^b^	0.00 ^c^	0.00 ^c^

^1^ MassBank; ^2^ [12]; ^3^ [13]; ^4^ [14]; ^5^ [15]; ^6^ [16]. ^abcde^ Values with different superscripts indicate significant difference among plant extracts (Tukey’s test; *p* < 0.05).

**Table 3:** Antioxidant activity determined by DPPH and ABTS methods (mg Trolox/100 g) of extracts obtained from the shells of different almond varieties.

	DPPH			ABTS		
Extract	Media		TD *	Media		TD
Constanti	100.13	±	11.14 ^c^	381.83	±	38.89 ^a^
D01-188	102.61	±	3.91 ^c^	323.37	±	34.78 ^ab^
Lauranne	197.97	±	29.69 ^a^	304.14	±	32.23 ^abc^
Marta	157.70	±	12.57 ^b^	234.78	±	28.95 ^c^
S4017	16.31	±	1.33 ^e^	127.85	±	24.79 ^d^
Vairo	61.10	±	2.62 ^d^	238.07	±	27.73 ^bc^

* TD: typical deviation; ^abcde^ mean values with different numbers indicate statistical differences (*p* < 0.05) between extracts.

**Table 4:** Antibacterial activity against different bacteria (% inhibition) of the different extracts obtained from the shells of different almond varieties.

	*B. cereus*			*S. aureus*			*E. coli*			*S. choleraesuis*			*L. innocua*			*L. monocytogenes*		
	Mean		TD *	Mean		TD	Mean		TD	Mean		TD	Mean		TD	Mean		TD
Extract (E)																		
Constanti	66.83	±	26.57 ^c^	81.97	±	12.3 ^b^	73.54	±	11.06 ^b^	97.11	±	3.85 ^a^	52.36	±	10.31 ^c^	53.71	±	25.32 ^cd^
D01-188	34.88	±	44.99 ^d^	41.71	±	26.99 ^d^	54.15	±	16.27 ^d^	85.90	±	14.11 ^b^	49.81	±	13.54 ^c^	42.89	±	32.86 ^d^
Lauranne	77.00	±	35.91 ^b^	64.83	±	45.69 ^c^	71.67	±	26.49 ^bc^	73.83	±	27.01 ^b^	75.50	±	23.76 ^b^	73.83	±	42.76 ^b^
Marta	34.00	±	50.37 ^d^	59.33	±	30.18 ^c^	66.33	±	17.59 ^bc^	85.00	±	19.80 ^b^	67.67	±	24.27 ^b^	66.17	±	24.36 ^bc^
S4017	99.37	±	1.13 ^a^	99.04	±	1.48 ^a^	98.20	±	3.10 ^a^	99.00	±	1.75 ^a^	98.08	±	0.93 ^a^	94.76	±	4.59 ^a^
Vairo	34.40	±	50.67 ^d^	85.58	±	20.82 ^b^	61.98	±	0.85 ^cd^	89.32	±	5.01 ^ab^	66.14	±	23.44 ^b^	50.62	±	37.42 ^d^
Concentration (C) (mg·L^−1^)																		
1600	97.42	±	3.63 ^a^	91.49	±	13.07 ^a^	77.75	±	19.67 ^a^	95.56	±	5.51 ^a^	86.18	±	17.27 ^a^	89.92	±	11.90 ^a^
800	47.87	±	45.05 ^b^	43.19	±	14.72 ^b^	70.85	±	19.62 ^a^	91.60	±	8.06 ^a^	64.36	±	19.71 ^b^	63.94	±	26.85 ^b^
400	27.94	±	37.34 ^c^	41.99	±	37.55 ^b^	64.33	±	19.71 ^b^	77.92	±	23.80 ^b^	54.24	±	21.86 ^c^	37.14	±	33.41 ^c^
*p* values																		
Pe	<0.001			0.044			<0.001			0.048			0.012			<0.001		
Pc	<0.001			0.038			<0.001			0.036			0.032			0.050		
Pe*c	<0.001			0.047			<0.001			0.025			0.062			0.009		

* TD: typical deviation; ^abcd^ mean values with different letters indicate statistical differences (*p* < 0.05) between factors.

**Table 5:** Growth (mm) of the tested mold strains obtained after 7 days of incubation at 25 °C in the presence of extracts obtained (at 5000 mg·L^−1^) from the shells of different almond varieties.

	*M. fructicola*			*B. cinerea*			*A. flavus*			*A. niger*			*P. expansum*		
Extract	Mean		TD *	Mean		TD	Mean		TD	Mean		TD	Mean		TD
Control	56.70	±	2.75	74.80	±	2.75 ^ab^	66.30	±	2.80 ^a^	80.90	±	1.39 ^a^	61.40	±	1.56 ^a^
Constanti	57.50	±	2.50	60.75	±	0.35 ^c^	52.50	±	0.71 ^b^	76.33	±	1.15 ^b^	48.17	±	0.29 ^b^
D01-188	60.83	±	2.89	80.00	±	2.00 ^ab^	65.50	±	0.71 ^a^	73.67	±	2.31 ^b^	49.83	±	1.26 ^b^
Lauranne	66.33	±	2.31	60.67	±	8.01 ^c^	60.83	±	2.89 ^a^	76.00	±	1.73 ^b^	51.00	±	7.47 ^ab^
Marta	64.67	±	4.51	71.17	±	4.65 ^ab^	61.00	±	2.60 ^a^	77.00	±	1.73 ^b^	61.67	±	6.29 ^a^
S4017	60.00	±	2.50	83.25	±	2.47 ^a^	61.00	±	1.41 ^a^	76.00	±	1.00 ^b^	44.50	±	7.26 ^b^
Vairo	60.83	±	10.41	70.17	±	6.81 ^bc^	54.67	±	0.58 ^b^	73.50	±	1.32 ^b^	48.50	±	2.12 ^b^

* TD: typical deviation; ^abc^ mean values with different letters indicate statistical differences (*p* < 0.05) between factors.

## Data Availability

Data availability statements are available in the relevant sections of this paper.
